# What Is the Difference between an Impulsive and a Timed Anticipatory Movement?

**DOI:** 10.1523/ENEURO.0322-25.2025

**Published:** 2025-11-11

**Authors:** Dominika Drążyk, Anissa Rida, Marcus Missal

**Affiliations:** Institute of Neurosciences (IONS), Cognition and System (COSY), Université catholique de Louvain, Brussels 1200, Belgium

**Keywords:** anticipation, eye movements, impulsivity, timing

## Abstract

Imagine yourself in a car race waiting for the traffic light to go green. Impulsivity could push you to accelerate when the light is still red. In contrast, temporally guided anticipation could lead you to accelerate at the time the light goes green. Whether these two types of early responses rely on the same or different neural processes is an open question. This question was investigated using an oculomotor task where the delay between a warning and an imperative visual stimuli was predictable. The spatial uncertainty of the “go” signal was also varied. On average, 10% of experimental trials were associated with a response before the “go” signal (“early saccade”). After the offset of the warning stimulus, the latency distribution of early saccades was bimodal, with a first mode peaking after 200 ms (1st mode saccades) and a second one starting to build-up after 375 ms (2nd mode saccades). With increasing delay duration: the number of 1st mode responses decreased whereas the number of 2nd mode responses remained approximately constant; the latency and variance of 2nd mode saccades increased; the maximum velocity of 2nd mode responses decreased. In general, the amplitude of 2nd mode responses was larger. These results show that there are probably two independent processes taking place before an expected event: an unintentional release of inhibition evoking an impulsive 1st mode saccade and an anticipatory process leading to a 2nd mode saccade.

## Significance Statement

Before an expected event, early motor responses are often observed. The cause of these responses during stimulus expectation is largely unknown. Two competing hypotheses have been suggested: lack of inhibition or anticipation. We studied early responses in an oculomotor task where temporal and spatial expectations were experimentally altered. Saccades during the expectation before the visual target could be categorized into two groups based on latency. The first group was composed of saccades that were not temporally guided, in contrast with saccades in the second group. Amplitude and maximum velocity of saccades in the second group were larger. Therefore, we show that two independent processes could be distinguished during the expectation of a visual stimulus: inhibitory control followed by temporal anticipation.

## Introduction

Imagine yourself in your car waiting for the traffic light to go from red to green. During this expectation period, sometimes one decides to accelerate when the light is still red. This behavior could be considered as resulting from a lack of “top-down” inhibitory control leading to an impulsive response. In contrast, it could also be considered as a cognitively driven anticipation allowing you to accelerate right at the time the light will go green and be slightly ahead. Therefore, there could be two different causes of early responses.

In an experimental environment, the equivalent of the car race is the “foreperiod paradigm.” The red signal is the warning stimulus (“WS”) whereas the equivalent of the green light is often referred to as the imperative stimulus (“IS”) or “go” signal. The delay between the “WS” and the “IS” is referred to as the foreperiod (“FP”) during which the expectation of the target builds up.

Several variants of the FP paradigm have been developed but usually based on a manual reaction time procedure (Niemi and Näätänen, 1981; Trillenberg et al., 2000; Los, 2013). More recently, saccadic eye movements were used as operant response (Janssen and Shadlen, 2005; de Hemptinne et al., 2008; Ameqrane et al., 2014; Hsu et al., 2019). Degos et al. (2022) used saccadic eye movements in a paradigm where a visual cue indicated the duration of the upcoming FP. This allowed optimal anticipation of the appearance of the visual “go” signal and a shortening of the latency of visually guided saccades. However, saccadic eye movements were also frequently observed during the FP, before the “go” signal with their latency forming a bimodal distribution. The 1st mode could be referred to as “impulsive” saccades (“false start”) and only 2nd mode responses were suggested to be “truly” anticipatory. Indeed, the timing of 1st mode responses was not changing with the duration of the cue in contrast with 2nd mode anticipatory saccades. Therefore, all responses before an expected stimulus cannot be qualified as “anticipatory” indiscriminately, as is usually the case. Instead, in Degos et al. (2022) it was suggested that responses before an imperative “go” stimulus should be collectively referred to as “early” movements, composed of both impulsive and anticipatory saccades.

Findings reported in Degos et al. (2022) suggest that there could be two different neural processes underlying early movements, with or without a cognitive top-down control of latency (referred to as the “two-process hypothesis”). However, these authors used a very particular paradigm where an explicit visual cue had to be associated with the upcoming FP duration. This association was rather abstract to learn. Furthermore, these authors did not investigate other saccade characteristics like amplitude and maximum velocity that could be different if 1st and 2nd mode saccades are the output of two different processes. Indeed, saccadic maximum velocity is a sensitive indicator of arousal and cognitive processing (Milstein and Dorris, 2007; DiStasi et al., 2013; Muhammed et al., 2020). Therefore, the maximum velocity of the eye could differ between 1st and 2nd mode saccades. In addition, saccade amplitude could reveal important information about the spatial locus of attention in the absence of a visual target but during its expectation.

The aim of the present study was to further test the two-process hypothesis. Therefore, we investigated the influence of spatial uncertainty and expected delay before the “go” signal on early saccades probability, latency, maximum velocity, and amplitude in an implicit timing task. If the two-process hypothesis were valid, then the amplitude and maximum velocity of 1st and 2nd mode saccades could be different at the beginning and end of the expectation period.

## Materials and Methods

### Subjects and ethics

Twenty-seven subjects participated in the present study. Two subjects were excluded due to extensive noise in oculomotor recordings resulting in a final sample of 25 subjects (18 women; mean ± SD, age 24.16 ± 3.72 years). Subjects were between 18 and 65 years old, had no known neurological or psychiatric diseases, did not take illegal psychoactive substances at least 1 d prior to the experiment, and had corrected to normal vision, if necessary. Each subject was informed about the aim of the study, signed an informed consent document regarding procedures, and was informed about the possibility of withdrawing from the experiment at any time without consequences. The study was carried out according to the Declaration of Helsinki guidelines and approved by the local Ethics Committee of the Université catholique de Louvain under number B403201733677 (Belgium).

### Experimental procedure

**Figure 1.**
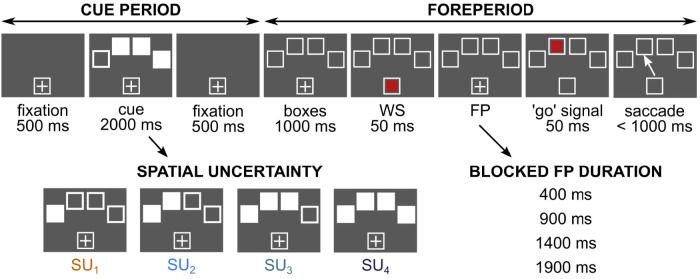
Schematic representation of the experimental design. Timeline of visual events on the screen in front of subjects. Each trial started with a fixation cross presented at the bottom of the screen in a white square box. During the cue period, while maintaining gaze on the fixation cross, subjects were presented with a spatial cue (cue boxes or “CB”) on top of the screen. CBs indicated the amount of expected spatial uncertainty bound to the future position of the target (SU_1_, SU_2_, SU_3_, or SU_4_). During the second part of the task, four empty test boxes (“TB”) were presented to subjects, followed by the warning signal (“WS”, the first red square) briefly presented in the lower box. The extinction of the WS initiated the foreperiod (“FP”). In a given block of trials, the FP could take one of four different durations (either 400, 900, 1,400, or 1,900 ms). At the end of the FP, the “go” signal (eccentric red square) was briefly presented in one of the four eccentric TBs. Subjects were asked to maintain their gaze on the fixation cross during the FP and then make a visually guided saccade toward the “go” signal as quickly as possible.

The task was composed of two different periods. During the cue period, spatial information was provided to subjects. During the second part of the task, the foreperiod paradigm was used. During the cue period, each trial started with the appearance of a white box (5.7 × 4.3° of visual angle) and a white fixation cross at the bottom of the screen for 500 ms (see Fig. 1 for details). Next, four cue boxes appeared for 2,000 ms (referred to as “CB”; 5.7 × 4.3° of visual angle each). The center of each cue box was at an approximate distance of 17° of visual angle from the center of the fixation box. From one to four CBs were uniformly white (Fig. 1, filled white squares). The number of filled white CBs cued subjects about spatial uncertainty in the upcoming foreperiod of the task. If the cue consisted of one marked box (*n* = 1, any of four presented boxes could be marked), the probability of the target appearing later in that box at the end of the foreperiod was P(TB_1_) = 1 (no uncertainty). If two boxes were marked (*n* = 2, two boxes had to be placed next to each other), the target could later appear in either one of them with the same probability P(TB_2_) = 0.5 (see Fig. 1 for details). If three boxes were marked (*n* = 3, boxes placed right next to one another), the probability for each box to be occupied by the future target decreased to P(TB_3_) = 0.33. Finally, if four boxes were marked (*n* = 4), the probability of each box to be occupied by the future target was P(TB_4_) = 0.25. Subjects were asked to maintain gaze on the fixation during the whole cue period.

During the second part of the task (foreperiod paradigm), four empty test boxes (“TB”, 5.7 × 4.3° of visual angle each) were displayed on the top of the screen for 1,000 ms at the same positions as the CBs. The warning signal (“WS”, red square) was shortly displayed in the fixation box for 50 ms. At the end of the FP, the “go” signal (eccentric red square) was displayed in one of the TBs for 50 ms. Subjects were instructed to maintain their gaze on the fixation cross during the FP and then make a visually guided saccade toward the “go” signal as fast as possible. In case a saccade occurred before the arrival of the “go” signal (further referred to as “early saccade”), the trial was not terminated and continued as planned. Each trial ended with an intertrial interval (“ITI”) of a randomized duration (2,250 ± 250 ms).

The experiment included four blocks of trials, each consisting of 120 trials. Each spatial cue (SU_1_, SU_2_, SU_3_, SU_4_) was randomly presented to the subject 30 times in each block, with 120 trials per cue overall. The duration of the FP was either 400, 900, 1,400, or 1,900 ms in a block of 120 trials. The order of different FP duration blocks was randomized between subjects.

The value of expected surprise (expected spatial uncertainty; in bits) bounded to each experimental condition (TB*_n_*) can be calculated using Shannon's formula (Shannon, 1948):

$$SU_{n}=-\log_{2}\;P(TB_{n}),$$

which is a negative base 2 logarithm of the probability of the target appearing on one of the marked boxes P(TB*_n_*). For example, in the TB_1_ condition, SU_1_ = 0 did not indicate any surprise about the outcome, and subjects were precisely informed about the box that would be occupied by the future “go” signal. The oculomotor preparation was maximal. The surprise for each condition was SU_1_ = 0, SU_2_ = 1, SU_3_ = 1.58, and SU_4_ = 2.

### Data collection and preprocessing

Eye movements were recorded binocularly at 500 Hz using an infrared eye tracking system (EyeLink 1000, desktop mount, SR Research) with an average spatial accuracy of 0.25° of visual angle and a pupil size resolution of 0.2% of diameter. The eye tracker was calibrated at the beginning of each data collection session and recalibrated every 30 trials after the subjects’ rest break. Subjects were sitting at a distance of 80 cm from the high-resolution screen (size: 54 × 30 cm, 1,920 × 1,080 pixels, VPixx Technologies), displaying stimuli at 60 Hz. Stimuli display and eye movement recordings were created using Experimental Builder (SR Research).

After the experiment, artifact rejection was performed with DataViewer (SR Research) to exclude oculomotor recordings of poor quality. Trials with frequent blinking, discontinuous recording, or drift of calibration were excluded from further analysis. Artifact rejection amounted to only 15% of all trials. The remaining pool of trials was searched by the saccade detection algorithm using amplitude (>1°), velocity (>22°/s), and acceleration (>3,800°/s^2^) criteria. Saccades occurring after the extinction of the WS and up to 100 ms after the “go” signal onset will be referred to as early saccades. The early saccades were detected in 10% of all trials remaining after artifact rejection. Saccades with a latency superior to or equal to 100 ms after the appearance of the “go” signal will be termed visually guided saccades (“VG”). The VG saccades with a latency >1,000 ms, executed after an early saccade or landing in the incorrect target box, were not considered for further analysis. The final pool of the trials with the VG saccades amounted to 78% of all trials remaining after artifact rejection.

Additionally, at the beginning of the experiment all participants filled up the self-administered Barratt Impulsivity Scale (Barratt, 1965; Patton et al., 1995) with the aim to assess their individual impulsivity levels through statements describing everyday situations.

### Statistical analysis

The parameters of early saccades were subjected to the LMM analysis. Each linear mixed-effects model used in the manuscript can be explained by the general equation:

$$Y_{is}∼\beta_{0}+\beta_{1}X_{is}+S_{0s}+\varepsilon_{is},$$

where *Y_is_* is the outcome for the *s*-th subject in the *i*-th trial, *X_is_* is the fixed effect for the *s*-th subject in the *i*-th trial, *β*_0_ is the intercept parameter, *β*_1_ is the slope parameter of the fixed effect, *S*_0*s*_ is the random intercept effect for subjects, and *ε_is_* is the corresponding residual error. First, to choose the best random effects structure, a “full” model (including all assumed variables) was fitted to data using the restricted maximum likelihood method (“REML”; Snijders and Bosker, 2011; Barr et al., 2013). The models used the following predictors: “FP”—the duration of the FP in the given block; “SU”—the level of expected spatial uncertainty. The different random structures of the compared full models were expressed using the “rs” suffix and the number: “full.rs1” describes the random intercept structure model that includes the ID of the subject as an intercept (“1 | subject”); “full.rs2” denotes the model with the random slope structure (“1 + SU | id”). The best random structure was chosen based on the lowest Bayes Information Criterion (“BIC”; Bolker et al., 2009). For the results of model selection, see Extended Data Figure 3-1. BIC was first calculated from models of different random effect structures, calculated using a REML method. Further, since more than one variable could influence the outcomes in this experiment, the best data-predicting model was chosen by refitting all comparable models using a maximum likelihood method (“ML”). Then, recalculated BIC values were compared. All continuous outcomes except early saccade count were fitted using the LMM Gaussian model of the *lme4* package (Bates et al., 2015) in R, with a chosen random structure. The main effects of each model were estimated using Type III ANOVA with Satterthwaite's method. The count of early saccades was fitted using the Poisson distribution within the generalized linear mixed models (“GLMM”) framework. If a large number of zeros in the measure was observed, a zero-inflated Poisson model was implemented using *glmmTMB* package (Brooks et al., 2017). The main effects of Poisson models were estimated using Type II Wald *χ*^2^ tests. The coefficients obtained from the GLMM analysis were expressed as incidence rate ratios (“IRR”) describing how much more likely is the occurrence of an event in one group compared with another. Pairwise comparisons for each dependent variable were calculated using the R package *emmeans* (Lenth, 2024). Diagnostics of each of the presented models were evaluated to ensure the quality of convergence and conformity with the assumptions. The quality of the model was assessed with the QQplot and residuals, using R packages *performance* (Lüdecke et al., 2021) and *DHARMa* (Hartig, 2022). The significance level assumed in the present study was *α* = 0.01.

Finally, we tested the possibility that the early saccade distribution was multimodal (Benaglia et al., 2009). A mixture of “*k*” Gaussian components was fitted to the distribution of early saccades, using the expectation-maximization algorithm [*normalmixEM* function, R package *mixtools* (Benaglia et al., 2009), maximal number of iterations: 1,000]. The *k* number ranged from 2 to 5 between mixtures (models *mix.2*, …, *mix.5*). As the FP duration changed between the experimental blocks, the mixture analysis was conducted on the early saccade distribution from each block, separately. The number of components that best fitted the data was determined by selecting the mixture with the lowest log-likelihood value (“LL”). The time point where the components intersected was then identified and further used to classify early saccades.

## Results

The present study focused on responses during the expectation period preceding the “go” signal or foreperiod. In a first approach, all responses occurring before the “go” signal will be referred to as “early responses.” For clarity, the influence of spatial uncertainty (SU) and foreperiod duration (FP) on early saccades will be presented separately.

### The influence of delay duration on early saccades latency and probability

**Figure 2.**
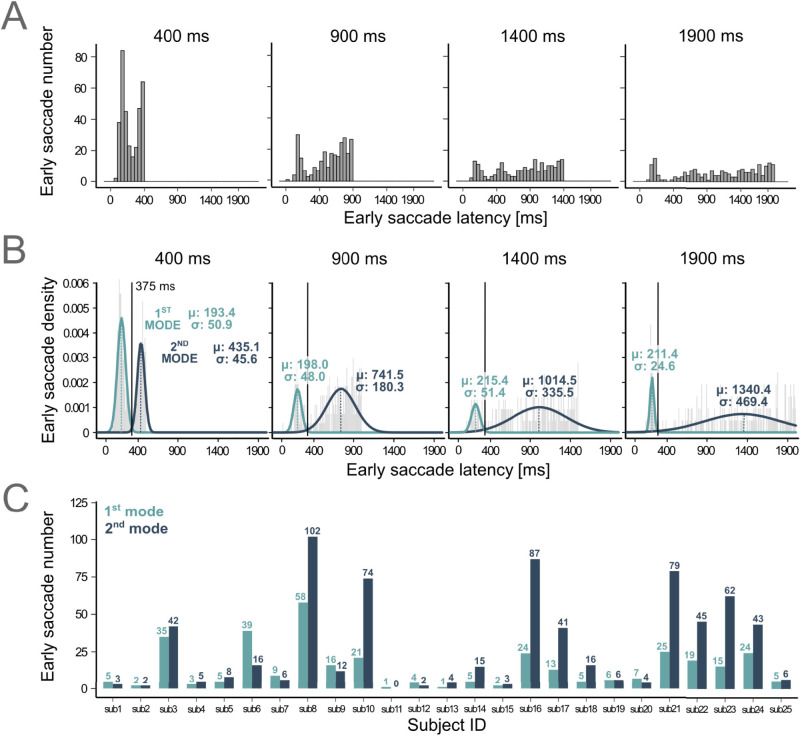
The latency distribution of early saccades. ***A***, Latency distributions of early saccades (bars) for each experimental condition. Time “zero” on the *x*-axis indicates the onset of the FP. ***B***, Latency distribution of early saccades (histograms) and results of the mixture models analysis (colored density plots) for the data presented in ***A***. A bimodal distribution consisting of a first and a second mode best described the data. Black dashed lines show the intersection between modes and the threshold value for the “cut” (375 ms, the same for all four FP blocks). Mean: *μ* (colored dashed lines); standard deviation: *σ*. ***C***, Number of early saccades for each subject and mode (colored bars). Labels show saccade counts.

In the present study, subjects were not informed of the temporal nature of the task and the timing of the “go” stimulus was implicit. The same FP duration was repeatedly presented in blocks of 120 trials. Figure 2*A* shows the latency distribution of early saccades for each FP duration tested. Visual inspection of the plots indicates that there was probably a 1st and 2nd mode for all distributions, as previously shown using an explicit timing cue (Degos et al., 2022). Therefore, in order to test the multimodality hypothesis of early saccade latency distributions and to determine the boundary between 1st and 2nd modes, a set of mixture models with different *k*, ranging between 1 and 5 was fitted to distributions for each FP duration. For all FP durations, the *mix.2* model assuming a bimodal distribution of early saccades latency fitted the data better (see Table 1 for details). The latency threshold to separate early saccades into two modes was arbitrarily set as the crossing point between the two Gaussian components. This approach yielded a constant crossing point for all FP durations tested (375 ms). Based on that threshold, there were 349 “1st mode saccades” and 760 “2nd mode saccades” collected (Fig. 2*B*). Individual counts of early saccades in both modes and for each subject are shown in Figure 2*C*.

**Table 1.** Log-likelihood of *k*-component mixture models fitted to the distribution of early saccades

FP duration	Log-likelihood			
	mix.2	mix.3	mix.4	mix.5
400 ms	−1,020.63	−1,011.12	−1,009.66	−1,008.95
900 ms	−938.54	−926.68	−927.72	−923.98
1,400 ms	−786.50	−775.42	−776.85	−782.52
1,900 ms	−794.67	−785.71	−784.53	−776.54

**Figure 3.**
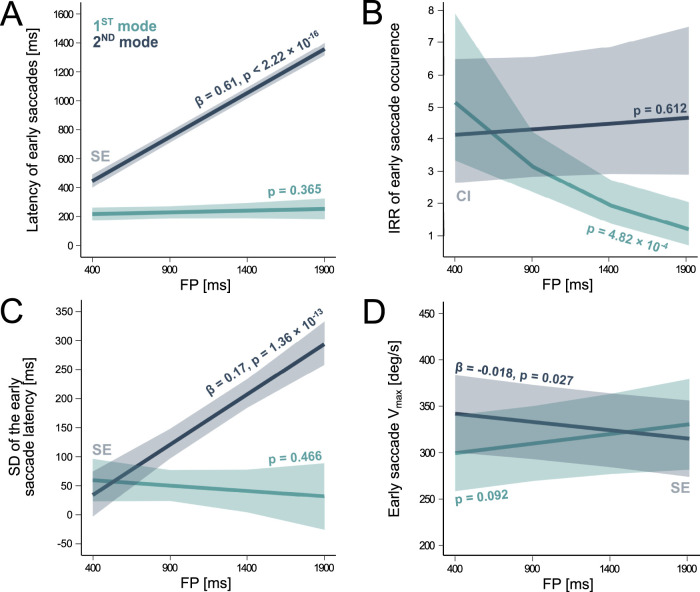
Characteristics of early saccades for different foreperiod durations. ***A***, Latency of the 1st and 2nd mode saccades as a function of FP duration. Colored regression lines describe post hoc LMM fits with the associated SE (ribbons). ***B***, Incidence Rate Ratio (“IRR”) of the 1st and 2nd mode saccades plotted against FP duration. Colored lines describe post hoc GLMM fits with the associated CI (ribbons). ***C***, SD of the 1st and 2nd mode saccade latencies plotted as a function of FP duration (colored regression line) with SE (colored ribbon). ***D***, *V*_max_ of 1st and 2nd mode saccades plotted against FP duration. Extended Data Figures 3-1–3-3 support this figure.

A visual assessment of the parameters of mixture components indicate that both the mean and the standard deviation of 2nd mode latencies increased with increasing FP duration (Fig. 2*B*). This is shown by comparing the trial-by-trial latencies of early saccades in 1st and 2nd mode as a function of increasing FP duration. With saccade latency as a dependent variable, we found a significant interactive effect between early saccade mode and FP duration (LMM, model *full.rs1*, *F*_(1,1031.98)_ = 366.28, *p* < 2.22 × 10^−16^; Extended Data Fig. 3-2). A post hoc analysis indicated that only the latency of 2nd mode saccades increased with FP duration (*β* = 0.61 ± 0.02, *p* < 2.22 × 10^−16^; Fig. 3*A*). In contrast, the latency of 1st mode saccades was not statistically increasing FP length (*p* = 0.365).

10.1523/ENEURO.0322-25.2025.f3-1 Figure 3-1 Random structure selection for the analysis of SU and FP duration effects on saccadic parameters. BIC values were derived from models fitted using the REML. Download Figure 3-1, DOCX file.

10.1523/ENEURO.0322-25.2025.f3-2 Figure 3-2 Influence of the FP duration and mode on the latency, V_max_ and amplitude of the early saccades. LMM models were fitted using the REML, statistics were calculated using Type III ANOVA. BIC values were derived from models re-fitted using the ML. Download Figure 3-2, DOCX file.

10.1523/ENEURO.0322-25.2025.f3-3 Figure 3-3 Influence of FP duration and mode on the count of early saccades. GLMM models were fitted using the ML, statistics were calculated using the Type III Wald Χ^2^ test. Download Figure 3-3, DOCX file.

It could be suggested that the probability of occurrence of a 1st or a 2nd mode saccade itself, independently of latency, could depend on FP duration. Using saccade number as dependent variable, a GLMM analysis indicated an interaction between the mode of early saccades and FP duration (model *full.rs4*, *χ*^2^_(1)_ = 11.67, *p* = 6.34 × 10^−4^; Extended Data Fig. 3-3). It was found that the incidence rate ratio (“IRR”) of 1st mode saccades decreased with FP duration (*β* = −0.001 ± 0.0003, *p* = 4.82 × 10^−4^). The occurrence of 1st mode saccades was ∼1.5 times more probable in a 400 ms FP compared with the 900 ms FP block (Fig. 3*B*). However, the rate of occurrence of 2nd mode saccades was not modified by FP length (*p* = 0.612).

Figure 2*B* also shows that the standard deviation of both modes of latency distributions could also covary with FP duration, particularly for 2nd mode saccades. An interaction between the mode of early saccades and FP duration on the standard deviation of latency distributions was found (LM, *F*_(1)_ = 34.68, *p* = 4.82 × 10^−8^). The SD of 1st mode of saccadic latencies was not modulated by FP length (*p* = 0.466). In contrast, the standard deviation of 2nd mode saccadic latencies increased with FP duration (*β* = 0.17 ± 0.02, *p* = 1.36 × 10^−13^; Fig. 3*C*).

### The influence of foreperiod duration on saccade kinematics

It was found that saccades *V*_max_ was higher for 2nd mode saccades (mean ± SE, 327.5 ± 20.3 deg/s) than 1st mode ones (306.3 ± 20.5 deg/s; diff = 21.2 ± 7.5 deg/s; model *mode.rs1*, *F*_(1,1015.27)_ = 8.04, *p* = 0.005; Extended Data Fig. 3-2) and an interaction effect between mode and FP duration was observed (model *full.rs1*, *F*_(1,1015.91)_ = 6.94, *p* = 0.009). The *V*_max_ of 1st mode saccades was approximately constant but it was decreasing with increasing FP duration for 2nd mode saccades (*β* = −0.02 ± 0.01, *p* = 0.027; Fig. 3*D*).

It was also found that the amplitude of 1st and 2nd mode saccades were different (model *mode.rs1*, *F*_(1,1013.92)_ = 18.95, *p* = 1.48 × 10^−4^; Extended Data Fig. 3-2). The amplitude of 2nd mode saccades (mean ± SE, 11.9 ± 0.5 deg) was on average 0.8 ± 0.2 deg larger than the amplitude of 1st mode saccades (11.1 ± 0.5 deg). However, no interaction between mode and FP duration was detected (model *full.rs1*, *p* = 0.112).

**Figure 4.**
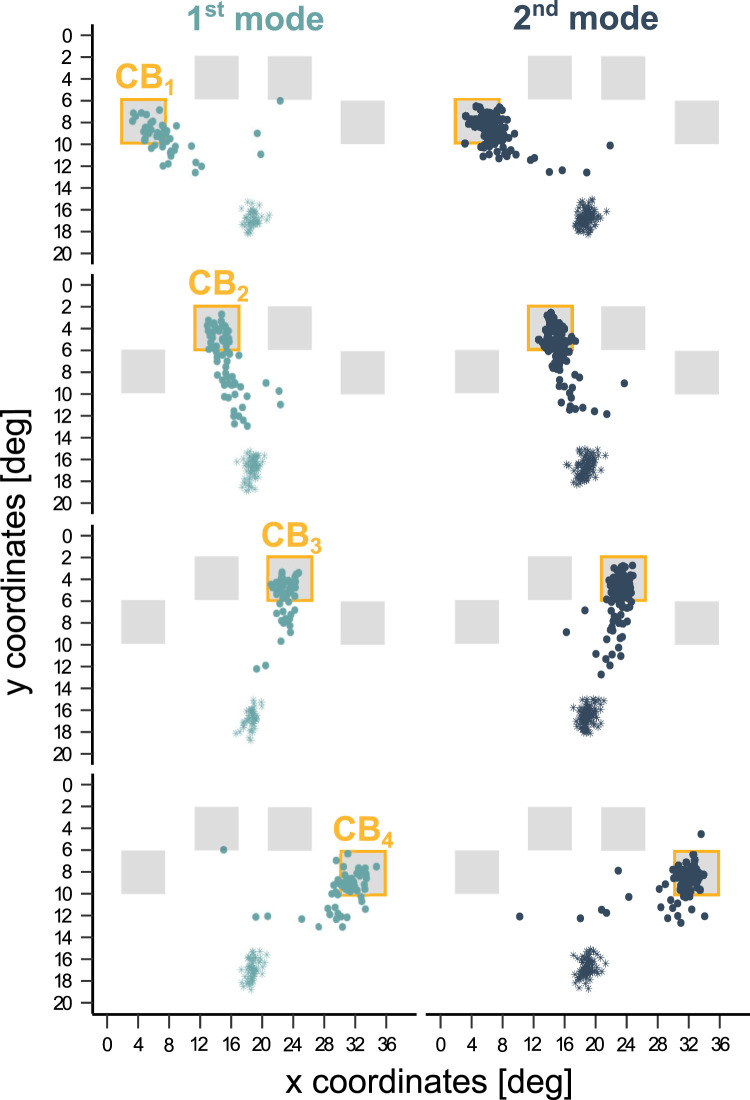
Trajectory of early saccades during the FP in the absence of spatial uncertainty (SU_1_). Starting (asterisks) and ending (dots) positions of early saccades, expressed in degrees of visual angle. Saccades were grouped by mode (columns) and depending on the localization of the cued box in the current trial (from CB_1_ to CB_4_, orange frame). Extended data Figures 4-1 and 4-2 support this figure.

Lastly, Figure 4 describes the trajectory of early saccades executed during the FP in the SU_1_ condition only. When the future localization of the incoming “go” signal was known, both the first and second mode saccades were directed at its anticipated position.

10.1523/ENEURO.0322-25.2025.f4-1 Figure 4-1 Influence of SU and mode on the count of early saccades. GLMM models were fitted using the ML, statistics were calculated using the Type III Wald Χ^2^ test. Download Figure 4-1, DOCX file.

10.1523/ENEURO.0322-25.2025.f4-2 Figure 4-2 Influence of SU and mode on latency, V_max_ and amplitude of early saccades. LMM models were fitted using the REML, statistics were calculated using Type III ANOVA. BIC values were derived from models re-fitted using the ML. Download Figure 4-2, DOCX file.

### The relationship between 1st and 2nd mode saccade occurrence across individuals

**Figure 5.**
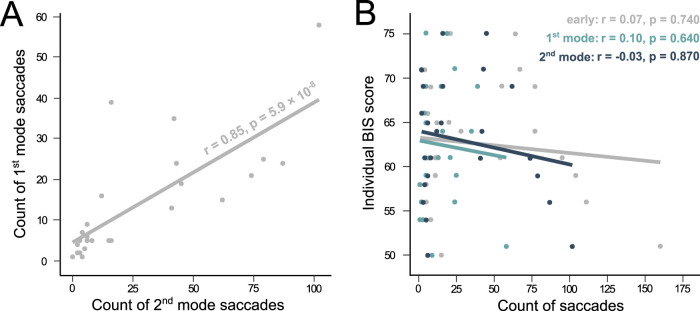
Individual early saccade counts. ***A***, Relationship between the count of 1st mode and 2nd mode saccades across subjects (dots). Spearman's rank correlation coefficient “*r*” is shown alongside the solid regression line. ***B***, Relationship between the count of early saccades (all early, 1st mode and 2nd mode) and individual Barratt Impulsivity Scale (“BIS”) score across subjects (*dots*). Spearman's rank correlation coefficient “*r*” is included in legend.

Figure 2*C* suggested a between-subjects correlation between the amount of 1st and 2nd mode saccades. Due to a violation of normality for the count distribution of 1st mode (Shapiro–Wilk normality test: *W* = 0.82, *p* = 0.0005) and 2nd mode saccades (*W* = 0.80, *p* = 0.0002), this analysis used the Spearman's rank correlation test. A significant positive correlation was found between those two measures (*r* = 0.85, *p* = 5.9 × 10^−8^) indicating that subjects who executed more 1st mode saccades also made more 2nd mode responses (Fig. 5*A*). Finally, a correlation analysis between the amount of early saccades and the Barratt Impulsivity Scale (“BIS”) scores between subjects was carried out. Due to a violation of normality of all early saccades count distribution (Shapiro–Wilk normality test: *W* = 0.83, *p* = 0.0008), all relationships were investigated using the Spearman's rank correlation test. No significant correlation was found between the individual BIS scores and the count distribution of 1st mode (*p* = 0.640), 2nd mode (*p* = 0.870), or all early saccades (*p* = 0.740; Fig. 5*B*).

### The influence of spatial uncertainty on early saccades

Based on Drążyk and Missal (2022), it was hypothesized that the number of occurrences of early responses could also be strongly affected by spatial uncertainty. Therefore, a GLMM analysis was applied to compare the number of early saccades for different values of SU including latency mode as a covariate. Spatial uncertainty significantly influenced the IRR of early saccades (*SU.rs2* model, *χ*^2^_(3)_ = 435.44, *p* < 2.22 × 10^−16^). Pairwise comparisons showed that the occurrence of early saccades was more likely in SU_1_ compared with the SU_2_ condition (IRR ± SE, 3.5 ± 0.3, *p* < 2.22 × 10^−16^), more likely in SU_1_ compared with the SU_3_ condition (3.5 ± 0.3, *p* < 2.22 × 10^−16^), and more likely in SU_1_ compared with the SU_4_ condition (4.5 ± 0.5, *p* < 2.22 × 10^−16^). However, there was no interaction between spatial uncertainty and mode of early saccades (*full.rs2* model, *p* = 480; Extended Data Fig. 4-1).

The *V*_max_ of early saccades was also influenced by spatial uncertainty (*SU.rs1* model, *F*_(3,1014.36)_ = 3.42, *p* = 0.017). Pairwise comparisons showed that the *V*_max_ of early saccades was higher in SU_1_ (*N* = 610, mean ± SE, 327.1 ± 20.1 deg/s) compared with the SU_3_ condition (*N* = 153, 293.1 ± 21.5 deg/s; diff = 34.0 ± 10.6 deg/s; *p* = 0.008). No interaction between spatial uncertainty and modality was found for the *V*_max_ of early saccades (*full.rs1* model, *p* = 0.150; Extended Data Fig. 4-2).

The amplitude of early saccades was also influenced by spatial uncertainty (*SU.rs1* model, *F*_(3,1015.08)_ = 37.81, *p* <  2.22 × 10^−16^). Pairwise comparisons showed that the amplitude of early saccades was 1.5 ± 0.2 deg higher in SU_1_ (*N* = 610; 13.1 ± 2.8 deg) compared with the SU_2_ condition (*N* = 160; 11.2 ± 3.3 deg; *p* = 1.33 × 10^−9^), 2.4 ± 0.3 deg higher in SU_1_ compared with the SU_3_ condition (*N* = 153; 9.9 ± 3.6 deg; *p* < 2.22 × 10^−16^), and 1.5 ± 0.3 deg higher in SU_1_ compared with the SU_4_ condition (*N* = 109; 10.7 ± 3.9 deg; *p* = 8.16 × 10^−7^). No interaction between spatial uncertainty and modality was found for the amplitude of early saccades (*full.rs1* model, *p* = 0.147; Extended Data Fig. 4-2).

Finally, the latency of early saccades was not influenced by spatial uncertainty (*SU.rs1* model, *p* = 0.861) and no interaction between spatial uncertainty and modality was found (*full.rs1* model, *p* = 0.987; Extended Data Fig. 4-2).

## Discussion

In the present study, we tested the two-process hypothesis during the expectation period preceding an imperative visual target. A blocked constant duration foreperiod design was used, and subjects were neither informed about the temporal nature of the task nor of the timing of the “go” signal. Furthermore, spatial uncertainty was varied on a trial-by-trial basis (Drążyk and Missal, 2022).

**Figure 6.**
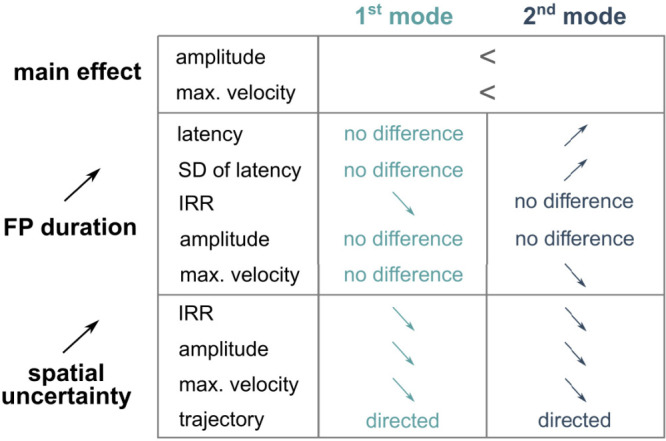
Characteristics of 1st and 2nd mode saccades in the context of increasing spatial uncertainty or increasing FP duration. Arrows pointing upward represent an increasing trend, while arrows pointing downward represent the decrease of a given saccadic parameter (first column). It can be observed that the influence of FP duration is what differentiates 1st from 2nd mode responses.

We confirm that the latency distribution of early saccadic responses was composed of two modes (Degos et al., 2022; Drążyk and Missal, 2022). It was found that the mean latency of 1st mode saccades was similar across FP durations but the latency of the 2nd mode saccades increased with FP duration (see summary of results in Fig. 6).

The variance of 2nd mode latencies only increased with FP duration (Fig. 6). In the temporal cognition literature, this is referred to as “scalar expectancy” (Gibbon, 1977). It has been hypothesized that scalar expectancy reflects the intentional use of temporal information in an explicit context, like comparison, estimation, or discrimination of durations (Buhusi and Meck, 2005; in the oculomotor domain, see Ameqrane et al., 2014). Here, it cannot be ruled out that through repetition of the same FP duration during several trials in a given block, an explicit representation could build up and guide 2nd mode responses, even if subjects were not previously informed about the temporal nature of the task. The absence of scalar expectancy on 1st mode saccades suggests that estimating the timing of the IS could require more processing time. When a 1st mode saccades was evoked, this processing could not have unfold due to the short latency of the response.

The number of 1st mode impulsive responses was decreasing with increasing FP duration, in contrast with the number of 2nd mode saccades that was approximately constant (Fig. 6). First mode saccades were more frequent for the 400 ms FP duration. We suggest that the shorter FP duration could have induced a confusion between the disappearance of the WS and the “go” signal, raising the probability of an impulsive 1st mode response. The approximately constant number of 2nd mode saccades suggests that the urge to anticipate the “go” target was constant but shifted toward the expected arrival time of the “go” signal.

Concerning movement kinematics, maximum eye velocity was higher for 2nd mode responses. Furthermore, the *V*_max_ of 1st mode responses was not influenced by FP duration, in contrast with 2nd mode ones whose velocity tended to decrease (Fig. 6). This could be interpreted as a reduction of alertness with elapsed time.

Increasing spatial uncertainty reduced the occurrence of all early responses similarly (Fig. 6). Therefore, spatial uncertainty could be causing a global top-down inhibition of all premotor processes before the “go” stimulus. This phenomenon could be similar to the nonselective response inhibition observed in go/no-go tasks with uncertainty about the go (or no-go) signal (Masharipov et al., 2022). Therefore, it could be suggested that uncertainty could be increasing inhibition in general, perhaps through the hyperdirect pathway between the frontal cortex and the subthalamic nucleus of the basal ganglia (Frank, 2006). Moreover, in the oculomotor domain, it has been shown that the supplementary eye fields (SEF) of the frontal cortex project to the nucleus raphe interpositus (nRIP) in the brainstem (Shook et al., 1988). Electrical stimulation of the so-called omnipause neurons of the nRIP interrupts eye movements. Therefore, the top-down oculomotor inhibition could come from the SEF region of the frontal cortex.

It could be suggested that impulsive 1st mode saccades are actually similar to “express” saccades frequently observed in the so-called oculomotor “gap” paradigm. In this paradigm, a central fixation target is firstly presented. After a constant duration delay referred to as the “gap” (typically 200 ms; Fischer and Ramspberger, 1984), an eccentric visual target is briefly presented in the periphery of the visual field. Subjects are instructed to wait for the eccentric target before making a visually-guided saccade. Average latency of these saccades is ∼150–200 ms. However, in a significant proportion of trials, a very short latency saccade (as short as 70 ms in the monkey) is initiated after the target appears (Boch et al., 1984; Weber et al., 1993). The latency distribution of express saccades is often clearly bimodal. Although 1st mode saccades occurred before target onset, their very short latency after the offset of the WS suggests that they could share some neural pathways. Express saccades are abolished after SC lesion, suggesting that this structure plays a central role in their generation (Schiller et al., 1987). We suggest that 1st mode saccades should be more affected by SC lesions than 2nd mode ones.

Altogether, results presented here support the hypothesis that 1st mode saccades could be classified as “impulsive” responses whereas 2nd mode saccades are intentional, cognitively driven responses but both were under top-down inhibition if SU increased. Therefore, it could be suggested that impulsive and anticipatory saccades are the outcome of two different neural processes but under a common inhibitory umbrella if there was spatial uncertainty. Early responses could be the expression of “waiting impulsivity” as is often described in the neuropsychiatric domain (Bari and Robbins, 2013). Therefore, we expected to find some correlation between psychometric scores of impulsivity (i.e., the BIS scale) and characteristics of early responses. However, no such correlation was found (Fig. 5*B*). Correlations between psychometric scales and behavioral responses are often tested with a mitigated success. In Cirilli et al. (2011), a correlation was found between the UPPS score and eye movement characteristics (urgency, lack of premeditation, lack of perseverance, and sensation seeking; Whiteside and Lynam, 2001). The absence of such a correlation in the present study could be related to the particular scale chosen or, more deeply, to a fundamental difference between waiting impulsivity and impulsivity as a character trait evaluated with a questionnaire. At the same time, it is worth noting that the correlation between the number of 1st and 2nd mode saccades suggests that the intensity of this top-down inhibition varies between subjects (Fig. 5*A*). This intensity could be further explored as a behavioral characteristic of “waiting impulsivity” useful in clinical evaluation.

## Synthesis

Reviewing Editor: Ifat Levy, Yale School of Medicine Decisions are customarily a result of the Reviewing Editor and the peer reviewers coming together and discussing their recommendations until a consensus is reached. When revisions are invited, a fact-based synthesis statement explaining their decision and outlining what is needed to prepare a revision will be listed below. The following reviewer(s) agreed to reveal their identity: Gustavo Rohenkohl. Note: If this manuscript was transferred from JNeurosci and a decision was made to accept the manuscript without peer review, a synthesis may not be available.

Reviewer comments:

I have read the authors' responses and the corresponding changes made to the manuscript. I am satisfied that they have adequately addressed my previous concerns, including clarifications about the correlation analyses and the revision of their neural interpretation, which has now been appropriately tone down. These revisions have strengthened the manuscript, and I now consider it suitable for publication.
