# Supplementary material for: What Is the Difference between an Impulsive and a Timed Anticipatory Movement?
Source: eNeuro. 2025 Nov 11;12(11):ENEURO.0322-25.2025. doi: 10.1523/ENEURO.0322-25.2025 (PMC12618049; doi:10.1523/ENEURO.0322-25.2025)
Supplement: Figure 3-1 — Random structure selection for the analysis of SU and FP duration effects on saccadic parameters. BIC values were derived from models fitted using the REML. Download Figure 3-1, DOCX file. [file eneuro-12-ENEURO.0322-25.2025-s002.docx]

### Figure 3-1 Random structure selection for the analysis of SU and FP duration effects on saccadic parameters. BIC values were derived from models fitted using the REML.

| *Outcome* | *Model* | *Formula* | *BIC (REML)* |
| --- | --- | --- | --- |
| early saccade latency | full.rs1 | latency ~ mode * FP + (1 \| subject) | 14203.47 |
|  | full.rs2 | latency ~ mode * FP + (1 + mode \| subject) | DNC |
|  | full.rs3 | latency ~ mode * FP + (1 + FP \| subject) | DNC |
|  | full.rs4 | latency ~ mode * FP + (1 + mode*FP \| subject) | DNC |
| early saccade count | full.rs1 | count ~ mode * FP + (1 \| subject) | 1001.016 |
|  | full.rs2 | count ~ mode * FP + (1 + mode \| subject) | 977.50 |
|  | full.rs3 | count ~ mode * FP + (1 + FP \| subject) | 980.48 |
|  | full.rs4 | count ~ mode * FP + (1 + mode * FP \| subject) | 959.95 |
| early saccade V_max_ | full.rs1 | V_max_ ~ mode * FP + (1 \| subject) | 12708.44 |
|  | full.rs2 | V_max_ ~ mode * FP + (1 + mode \| subject) | DNC |
|  | full.rs3 | V_max_ ~ mode * FP + (1 + FP \| subject) | DNC |
|  | full.rs4 | V_max_ ~ mode * FP + (1 + mode * FP \| subject) | DNC |
| early saccade amplitude | full.rs1 | amp ~ mode * FP + (1 \| subject) | 5088.86 |
|  | full.rs2 | amp ~ mode * FP + (1 + mode \| subject) | DNC |
|  | full.rs3 | amp ~ mode * FP + (1 + FP \| subject) | 5101.63 |
|  | full.rs4 | amp ~ mode * FP + (1 + mode * FP \| subject) | DNC |
| early saccade latency | full.rs1 | latency ~ mode * SU + (1 \| subject) | 15064.02 |
|  | full.rs2 | latency ~ mode * SU + (1 + mode \| subject) | DNC |
|  | full.rs3 | latency ~ mode * SU + (1 + SU \| subject) | DNC |
|  | full.rs4 | latency ~ mode * SU + (1 + mode*SU \| subject) | DNC |
| early saccade count | full.rs1 | count ~ mode * SU + (1 \| subject) | 940.13 |
|  | full.rs2 | count ~ mode * SU + (1 + mode \| subject) | 909.53 |
|  | full.rs3 | count ~ mode * SU + (1 + SU \| subject) | DNC |
|  | full.rs4 | count ~ mode * SU + (1 + mode * SU \| subject) | DNC |
| early saccade V_max_ | full.rs1 | V_max_ ~ mode * SU + (1 \| subject) | 12671.65 |
|  | full.rs2 | V_max_ ~ mode * SU + (1 + mode \| subject) | DNC |
|  | full.rs3 | V_max_ ~ mode * SU + (1 + SU \| subject) | DNC |
|  | full.rs4 | V_max_ ~ mode * SU + (1 + mode * SU \| subject) | DNC |
| early saccade amplitude | full.rs1 | amp ~ mode * SU + (1 \| subject) | 5005.44 |
|  | full.rs2 | amp ~ mode * SU + (1 + mode \| subject) | 5012.19 |
|  | full.rs3 | amp ~ mode * SU + (1 + SU \| subject) | 5050.36 |
|  | full.rs4 | amp ~ mode * SU + (1 + mode * SU \| subject) | DNC |

*DNC* model did not converge.
