# Supplementary material for: What Is the Difference between an Impulsive and a Timed Anticipatory Movement?
Source: eNeuro. 2025 Nov 11;12(11):ENEURO.0322-25.2025. doi: 10.1523/ENEURO.0322-25.2025 (PMC12618049; doi:10.1523/ENEURO.0322-25.2025)
Supplement: Figure 3-2 — Influence of the FP duration and mode on the latency, Vmax and amplitude of the early saccades. LMM models were fitted using the REML, statistics were calculated using Type III ANOVA. BIC values were derived from models re-fitted using the ML. Download Figure 3-2, DOCX file. [file eneuro-12-ENEURO.0322-25.2025-s003.docx]

### Figure 3-2 Influence of the FP duration and mode on the latency, V_max_ and amplitude of the early saccades. LMM models were fitted using the REML, statistics were calculated using Type III ANOVA. BIC values were derived from models re-fitted using the ML.

| *Outcome* | *Model* | *BIC (ML)* | *Fixed terms* | *df* | *F value* | *p value* | *Random terms 𝜎* | |
| --- | --- | --- | --- | --- | --- | --- | --- | --- |
|  |  |  |  |  |  |  | *subject* | *resid* |
| Saccadic latency | full.rs1 | 14203.47 | mode | 1, 1031.33 | 0.016 | 0.900 | 70.77 | 227.43 |
|  |  |  | FP | 1, 1032.00 | 424.41 | < 2.2 × 10^-16^ |  |  |
|  |  |  | mode * FP | 1, 1031.98 | 366.27 | < 2.2 × 10^-16^ |  |  |
|  | mode.rs1 | 15027.27 | mode | 1, 1031.70 | 913.06 | < 2.2 × 10^-16^ | 93.23 | 341.94 |
|  |  |  |  |  |  |  |  |  |
|  | FP.rs1 | 15018.98 | FP | 1, 1028.09 | 926.99 | < 2.2 × 10^-16^ | 136.97 | 338.44 |
|  |  |  |  |  |  |  |  |  |
| Saccadic V_max_ | full.rs1 | 12707.33 | mode | 1, 1013.80 | 14.557 | 1.44 × 10^-4^ | 94.63 | 108.23 |
|  |  |  | FP | 1, 1014.83 | 0.036 | 0.850 |  |  |
|  |  |  | mode * FP | 1, 1015.91 | 6.937 | 0.009 |  |  |
|  | mode.rs1 | 12701.23 | mode | 1, 1015.27 | 8.036 | 0.005 | 95.05 | 108.53 |
|  |  |  |  |  |  |  |  |  |
|  | FP.rs1 | 12709.20 | FP | 1, 1015.58 | 0.057 | 0.811 | 95.98 | 108.93 |
| Saccadic amplitude | full.rs1 | 5088.86 | mode | 1, 1012.85 | 16.787 | 0.038 | 2.13 | 2.71 |
|  |  |  | FP | 1, 1014.36 | 4.334 | 4.51 × 10^-5^ |  |  |
|  |  |  | mode * FP | 1, 1015.64 | 2.535 | 0.112 |  |  |
|  | mode.rs1 | 5086.17 | mode | 1, 1013.92 | 18.941 | 1.48 × 10^-5^ | 2.20 | 2.72 |
|  | FP.rs1 | 5103.88 | FP | 1, 1014.67 | 1.061 | 0.303 | 2.18 | 2.74 |

*df* degrees of freedom, *resid* residual, *𝜎* SD of the random terms.
