# Supplementary material for: What Is the Difference between an Impulsive and a Timed Anticipatory Movement?
Source: eNeuro. 2025 Nov 11;12(11):ENEURO.0322-25.2025. doi: 10.1523/ENEURO.0322-25.2025 (PMC12618049; doi:10.1523/ENEURO.0322-25.2025)
Supplement: Figure 3-3 — Influence of FP duration and mode on the count of early saccades. GLMM models were fitted using the ML, statistics were calculated using the Type III Wald Χ2 test. Download Figure 3-3, DOCX file. [file eneuro-12-ENEURO.0322-25.2025-s004.docx]

### Figure 3-3 Influence of FP duration and mode on the count of early saccades. GLMM models were fitted using the ML, statistics were calculated using the Type III Wald 𝛸^2^ test.

| *Model* | *BIC (ML)* | *Fixed terms* | *df* | 𝛸^2^ *value* | *p value* | *Random terms 𝜎* |
| --- | --- | --- | --- | --- | --- | --- |
|  |  |  |  |  |  | *subject* |
| full.rs4 | 959.95 | mode | 1 | 1.186 | 0.276 | 0.57 |
|  |  | FP | 1 | 1.150 | 0.284 |  |
|  |  | mode * FP | 1 | 11.672 | 6.34 × 10^-4^ |  |
| mode.rs4 | 959.39 | mode | 1 | 0.470 | 0.493 | 0.52 |
|  |  |  |  |  |  |  |
| FP.rs4 | 959.47 | FP | 1 | 0.385 | 0.535 | 0.51 |
|  |  |  |  |  |  |  |

df degrees of freedom, 𝜎 SD of the random terms.
