# Supplementary material for: What Is the Difference between an Impulsive and a Timed Anticipatory Movement?
Source: eNeuro. 2025 Nov 11;12(11):ENEURO.0322-25.2025. doi: 10.1523/ENEURO.0322-25.2025 (PMC12618049; doi:10.1523/ENEURO.0322-25.2025)
Supplement: Figure 4-1 — Influence of SU and mode on the count of early saccades. GLMM models were fitted using the ML, statistics were calculated using the Type III Wald Χ2 test. Download Figure 4-1, DOCX file. [file eneuro-12-ENEURO.0322-25.2025-s005.docx]

### Figure 4-1 Influence of SU and mode on the count of early saccades. GLMM models were fitted using the ML, statistics were calculated using the Type III Wald 𝛸^2^ test.

| *Model* | *BIC (ML)* | *Fixed terms* | *df* | 𝛸^2^ *value* | *p value* | *Random terms 𝜎* |
| --- | --- | --- | --- | --- | --- | --- |
|  |  |  |  |  |  | *subject* |
| full.rs2 | 909.53 | mode | 1 | 5.141 | 0.023 | 0.82 |
|  |  | SU | 3 | 434.217 | < 2.2 × 10^-16^ |  |
|  |  | mode * SU | 3 | 2.473 | 0.480 |  |
| mode.rs2 | 1318.63 | mode | 1 | 3.955 | 0.047 | 0.69 |
|  |  |  |  |  |  |  |
| SU.rs2 | 896.32 | SU | 3 | 435.44 | < 2.2 × 10^-16^ | 0.77 |
|  |  |  |  |  |  |  |

*df* degrees of freedom, *𝜎* SD of the random terms.
